# Nodular Lymphocyte-Predominant Hodgkin Lymphoma in Progressive Transformation of Germinal Centers

**DOI:** 10.1155/2017/5982168

**Published:** 2017-11-14

**Authors:** C. Eric Bailey, Francesca Jung, Benjamin Addicks, Olukemi A. Esan, Brian Kellermeyer

**Affiliations:** ^1^Department of Otolaryngology, West Virginia University Health Science Center, Morgantown, WV, USA; ^2^School of Medicine, West Virginia University, Morgantown, WV, USA; ^3^Department of Pathology, West Virginia University Health Science Center, Morgantown, WV, USA

## Abstract

Nodular lymphocyte-predominant Hodgkin lymphoma is an uncommon variant of Hodgkin lymphoma. Progressive transformation of germinal centers has been associated with and can develop prior to, concurrent with, or after the diagnosis of nodular lymphocyte-predominant Hodgkin lymphoma. We present a patient with a history of progressive transformation of germinal centers of the right parotid who presented 4 years later with ipsilateral parotid mass and cervical adenopathy. Knowledge of her previous diagnosis raised our concern for lymphoma, influenced our surgical management, and spared the patient additional surgery with risk of facial nerve injury inherent in revision parotidectomy.

## 1. Introduction

Masses of the parotid gland warrant thorough investigation, as 15–32% of parotid gland masses are found to be malignant [1]. The course of malignant parotid gland tumors or other salivary gland tumors is highly variable, but they have the potential to be aggressive and result in high levels of morbidity and mortality. Painless swellings of the parotid gland, especially those involving the facial nerve, necessitate pathologic examination with either fine-needle aspiration (FNA) or ultrasound-guided core needle biopsy. FNA, in particular, has been found to be highly specific in determining benign versus malignant parotid gland lesions [2] and is strongly recommended when the mass could possibly represent mimickers such as TB, lymphoma, or enlarged lymph nodes [1].

Alternatively, progressive transformation of germinal centers (PTGC) typically presents as an asymptomatic enlarged lymph node of the head and neck [2]. First reported by Lennert and Müller-Hermelink, PTGC is a benign condition that can be mistaken for malignant processes such as nodular lymphocyte-predominant Hodgkin lymphoma (NLPHL) and follicular lymphoma [3]. The name itself somewhat describes the entity; the germinal center expands as mantle zone lymphocytes infiltrate the germinal center and blur the margin between the mantle zone and germinal center [2, 4]. Although the pathogenesis of PTGC is unclear and likely multifactorial, it is believed to be a state of follicular hyperplasia following reactive stimuli [5, 6]. PTGC and nodular lymphocyte-predominant Hodgkin lymphoma (NLPHL) are often found concurrently, or with one entity preceding the other. While PTGC may precede NLPHL, some studies have shown that patients with PTGC did not show an increased risk for developing NLPHL, making it uncertain whether the clonal population of cells comprising NLPHL arises from PTGC directly [2, 6].

PTGC presents most often in males with a 3 : 1 male to female ratio and generally presents as painless, persistent lymphadenopathy [7]. Cervical chain lymph nodes are most frequently affected by PTGC, with nearly 50% of cases, followed by inguinal (25%) and axillary (22%) [1]. Approximately 3.5–10% of patients presenting with painless, persistent lymphadenopathy are diagnosed with PTGC [8]. In prior reports, anywhere between 16 and 35% of PTGC are associated with NLPHL [7, 9, 10].

We present a case of a 39-year-old female patient in which the knowledge of prior diagnosis of PTGC influenced workup and management when she presented with recurrent parotid mass and ipsilateral lymphadenopathy.

## 2. Case Report

A 39-year-old female presented to the clinic with right neck lymphadenopathy and an apparent ipsilateral parotid mass. Her previous history was significant for ipsilateral superficial parotidectomy and neck dissection performed at another institution. Per the patient, the pathology report from her parotidectomy showed no lymphoma or other malignancy, but she did mention that she was sent to an oncologist after surgery. After a four-year symptom-free interval, she presented to us with recurrent right parotid mass, neck lymphadenopathy, fatigue, and weight loss. Facial nerve was intact bilaterally.

Given the recurrent nature, presence of lymphadenopathy, and the patient's age, suspicion of malignancy was high. Multiple fine-needle aspiration biopsies of the cervical lymph nodes and parotid mass failed to establish diagnosis. When pathology was obtained from her previous surgery, the report described PTGC. After review of the pathology from the previous procedure, the decision was made to proceed with excisional biopsy of level II cervical lymph node, sparing the previously operated parotid space with inherent risk of facial nerve injury in revision parotidectomy. Pathology revealed NLPHL in a portion of the node, adjacent to areas of PTGC.

The patient had an uncomplicated postoperative recovery without facial nerve injury or other morbidities. Postoperative PET/CT showed hypermetabolic lymphadenopathy of the right neck, parotid, and right inguinal areas. She was treated with chemotherapy with good response to treatment.

## 3. Pathology Discussion

It is well known that progressive transformation of germinal centers can mimic nodular lymphocyte-predominant Hodgkin lymphoma both clinically and histologically [3]. While PTGC may be a precursor of NLPHL, many patients with PTGC do not develop NLPHL later in life [11]. Of note, PTGC may be present in the same lymph node as NLPHL; thus, careful sampling is required to exclude malignancy in lymph nodes involved by PTGC [12]. Progressively transformed follicles are similar to NLPHL nodules in that they are composed of nonneoplastic small mantle B cells. However, interspersed hyperplastic follicles are typically present, which is not usual in NLPHL [3]. In addition, the nodules do not contain the neoplastic lymphocyte-predominant (LP) cells seen in NLPHL. These LP cells are larger atypical lymphocytes with lobulated nuclei resembling kernels of popped corn also referred to as popcorn cells [13]. By immunohistochemical staining, the LP cells express CD20 and CD45 while being negative for CD15 and CD30. In addition, PD-1-positive T cells form rosettes around LP cells in NLPHL [13].

## 4. Discussion

A parotid mass with lymphadenopathy in an adult patient necessitates evaluation. While FNA is commonly employed in the workup of salivary lesions or lymphadenopathy, it is often nondiagnostic when dealing with hematogenous malignancies, with core biopsy or excisional biopsy often needed to provide adequate sample for analysis of node architecture and diagnosis. This particular patient presented with worrisome progressive enlargement, weight loss, and concern for malignancy. At the initial clinic encounter when pathology from the prior surgery was unavailable, the lymphadenopathy prompted concern for carcinoma *ex pleomorphic* (given her previous parotidectomy), metastatic squamous cell carcinoma, mucoepidermoid carcinoma, or some other primary salivary malignancies. After previous pathology was obtained, the noted association between PTGC and NLPHL was considered when FNA as a diagnostic modality failed. This influenced surgical decision-making and led us to undertake excisional biopsy of a cervical node rather than parotidectomy. Based on frozen section which suggested hematologic malignancy was most likely, this spared the patient an extensive revision parotidectomy and the associated morbidity of such a surgery.

**Figure 1:**
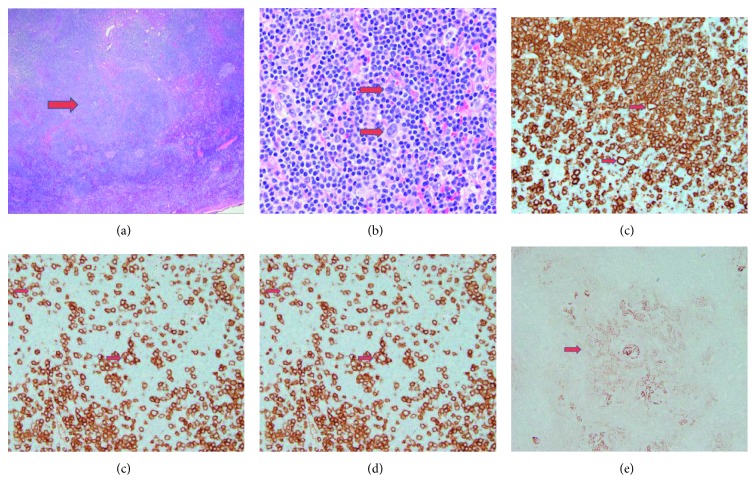
(a) Low-power H&E staining showing prominent nodules in the background of reactive lymphoid tissue. (b) Interspersed large “popcorn” lymphocyte-predominant cells. (c) Large popcorn cells are positive for CD20. (d) CD3-positive T cells form rosettes around the large popcorn cells. (e) CD21 staining showing expanded follicular dendritic network in the large nodule.

In a thorough English language literature search, the percentage of cases of progressive transformation of germinal centers associated with nodular lymphocyte-predominant Hodgkin lymphoma was 25.1%, as shown in Figure 1. The diagnosis of NLPHL could precede, antecede, or appear concurrently with a diagnosis of PTGC. Prior reports found the association to be between 16 and 35% [7, 11]. A PTGC diagnosis prior to a diagnosis of NLPHL, as in the case of our patient, is rare. In our literature review, a total of 9 reported cases out of 275 (3.27%) were found to have PTGC preceding diagnosis of NLPHL. However, the association overall is sufficient to consider following patients with either PTGC or NLPHL for development of the alternative disease. Additionally, as the rate of concurrent PTGC and NLPHL has the strongest association, it is important to be certain of a single diagnosis when either is discovered. A diagnosis of NLPHL succeeding PTGC may appear anywhere from less than one year to 13 years after the diagnosis of PTGC, and the fear of diagnosis may lead to multiple repeat biopsies [14]. Although our patient's NLPHL was found in the same location as her prior PTGC, NLPHL may be found in separate lymph nodes, most commonly of the cervical chain [7]. There is no consensus at this point regarding the appropriate follow-up interval or imaging for PTGC. Further study should be considered to evaluate the proper interval and duration of follow-up for patients with PTGC (Table 1).

## Figures and Tables

**Table 1:** Summary of literature.

Study	Year of publication	Cases of PTGC	NLPHL diagnosis, total	PTGC before NLPHL	Concurrent	NLPHL before PTGC
Poppema et al. [15]	1979	5	5	2	1	2
Osborne and Butler [6]	1984	50	12	0	5	7
Burns et al. [12]	1984	36	36	2	31	3
Crossley et al. [16]	1987	1	1	1	0	0
Hansmann et al. [11, 17]	1990	66	11	3	4	4
Ferry et al. [18]	1992	5	0	0	0	0
Verma et al. [19]	2002	2	0	0	0	0
Kojima et al. [2]	2003	42	0	0	0	0
Licup et al. [20]	2006	5	0	0	0	0
Shaikh et al. [14]	2012	29	1	1	0	0
Chang et al. [9]	2015	1	0	0	0	0
Özkan et al. [10]	2016	33	3	0	0	1

Total		275	69	9	43	17
